# The Impact of Virtual-, Augmented- and Mixed Reality during Preoperative Informed Consent: A Systematic Review of the Literature

**DOI:** 10.1007/s10916-025-02217-9

**Published:** 2025-06-24

**Authors:** Konstantin Wehrkamp, Rainer C. Miksch, Hans Polzer, Fabian Gilbert, Markus Bühner, Boris M. Holzapfel, Wolfgang Böcker, Rouven Neudeck

**Affiliations:** 1 Department of Orthopaedics and Trauma Surgery, Musculoskeletal University Center Munich (MUM), University Hospital, LMU Munich, 81377 Munich, Germany; 2 Department of Psychology, LMU Munich, 80802 Munich, Germany; 3 OrthoPlus, Lehnbachplatz 2a, Munich, 80333 Germany

**Keywords:** 3D virtual reality, Communication, Head-mounted display, Patient education, Patient engagement, Preoperative informed consent, Surgery, Surgical interventions

## Abstract

This systematic literature review aimed to examine the effects of Virtual Reality (VR), Augmented Reality (AR), and Mixed Reality (MR) head-mounted displays (HMDs) on patient understanding, satisfaction, and anxiety during preoperative informed consent. Following PRISMA-P guidelines (Prospero ID: CRD42023487281), we searched four major databases from their inception to March 24, 2023. Studies were eligible if they utilized VR, AR, or MR HMDs to visualize patient-specific data during informed consent across any medical specialty. Two reviewers independently conducted all steps of the systematic review process, and the risk of bias was assessed using the Methodological Index for Non-Randomized Studies (MINORS). Sixteen studies involving a total of 1067 patients were identified and included. These comprised 10 Randomized Controlled Trials (RCTs) and 6 Non-Randomized Controlled Trials (non-RCTs), including one comparative study and five non-comparative studies. The literature reviewed was heterogeneous, encompassing patients with diverse conditions across various medical specialties, including cardiology, neurosurgery, transplantation surgery, vascular surgery, plastic surgery, and urology. The results demonstrated that VR, AR, and MR HMDs positively impact patient understanding, satisfaction, and anxiety reduction. Notably, the findings were more consistent for VR HMDs compared to the limited and variable literature on AR and MR HMDs. VR, AR, and MR HMDs generally show positive effects on patient understanding, satisfaction, and anxiety in preoperative informed consent. While VR HMDs consistently yield positive outcomes, further research is needed to elucidate the effectiveness and benefits of AR and MR HMDs in preoperative consultations.

## Introduction

Preoperative surgical informed consent is crucial for establishing strong patient relationships, often presenting complex challenges. It involves providing patients with essential information about the risks and benefits of treatments, genetic testing, or clinical trials, enabling them to make informed decisions. This process is grounded in the ethical principle of patient autonomy, ensuring decisions are made based on a thorough understanding of their medical situation [1].

A key aspect of informed consent is shared decision-making, where physicians offer evidence-based and experience-based treatment recommendations [2]. This collaborative approach often involves deciding whether to treat an illness conservatively or surgically, with the patient ultimately maintaining autonomy in their choice. Studies indicate that patients generally prefer to take responsibility for their decisions, and increased involvement leads to better health outcomes [2–5].

To make informed decisions, patients need a comprehensive understanding of their condition. Radiological imaging, such as X-rays or CT scans of fractures, helps in this process. For clarity, these images must be presented in a way that is accessible and easy to comprehend. Traditional methods may be insufficient for patients without medical backgrounds, often leading to misunderstandings and anxiety.

Innovative visualization methods, including Virtual Reality (VR), Augmented Reality (AR), and Mixed Reality (MR), can be utilized to present individualized radiological imaging. VR head-mounted displays (HMDs) provide full immersion, while AR and MR overlay virtual objects, such as 3D holograms, onto the real environment [6]. The distinction between AR and MR varies in the literature; some consider MR synonymous with AR, while others suggest that MR simplifies the interaction between the virtual and real worlds [7–9]. Consequently, patients undergoing a wide variety of surgical procedures may benefit from the use of these innovative technologies, particularly in cases where visualizing the surgical intervention is a key factor in facilitating shared decision-making.

In the medical field, VR, AR, and MR HMDs are used pre- and intraoperatively for planning and as surgical aids [10–12], and also serve educational purposes for medical staff and patients [11–13]. Studies indicate that 3D reconstructions or virtual “magic mirrors” enhances patients’ understanding of diseases compared to standard X-ray, CT, or MRI images [14–17]. has also been shown to reduce anxiety during anaesthesia induction, enhance parental comprehension, and increase surgical preparedness in children and their families [18–21].

Despite these benefits, routine clinical use of these technologies for informed consent remains limited due to challenges such as technical barriers, costs, and the need for training. Additionally, inconsistent study quality highlights the need for more robust research.

This systematic review aims to summarize current evidence on the use of VR, AR, and MR HMDs in preoperative education for adult surgical patients and their impact on informed consent. By synthesizing data from a broad range of studies, this review seeks to elucidate the benefits and limitations of these advanced visualization technologies in enhancing patients’ understanding and satisfaction and in reducing anxiety. Furthermore, it aims to identify gaps in the existing literature and suggest directions for future research to optimize the use of VR, AR, and MR in clinical settings.

## Materials and Methods

This systematic literature review was conducted in accordance with PRISMA guidelines. The study has been registered with PROSPERO under the identifier CRD42023487281 [22].

### Literature Search

The PICOS strategy was determined as shown in Table 1.

**Table 1:** PICOS (population, intervention, comparison, outcome, study design) strategy of the study

	Description	Inclusion	Exclusion
Population	Adults needing surgery	≥ 18 years	< 18 years, parents of patients
Intervention	Preoperative patient education with VR, AR, or MR HMDs	HMDs must be glasses	Non-glasses HMDs (e.g. phone holders)
Comparison	Preoperative patient education with other tools	Non-HMD tools (e.g., verbal, paper, X-ray)	No exclusion
Outcome	Impact on patients’ understanding, satisfaction, and anxiety	Subjective and objective data from patients	No concrete outcome
Study design	Studies in English/German, published by March 24, 2023	RCTs, Non-RCTs, prospective trials, case series (≥ 5 patients)	Studies < 5 patients, reviews, meta-analyses, non-English/German, published after March 24, 2023

A systematic search was conducted across four electronic databases: Medline (PubMed), Embase, Scopus, and Central. The search strategy, implemented on March 24, 2023, comprised three principal components connected using the Boolean operator “AND”. The first component focused on visualization methods (VR, AR, or MR), the second component linked these methods to surgical procedures, and the third component addressed patient education and patient satisfaction. To minimize the risk of excluding relevant studies, various synonyms were employed for each search term. The search strategy was tailored to meet the specific requirements of each database.

Additionally, the references of identified reviews and studies were examined for potential inclusion. Studies identified through this manual review that were not captured in the electronic search, were also included. Corresponding authors of papers were contacted by the author (KW) for missing data or necessary clarifications. Detailed information on the search strategy is provided in the Appendix.

### Study Selection and Data Extraction

The literature search was conducted separately across the selected databases, and the findings were exported to EndNote™ (version 21.4; Clarivate). After removing duplicates, the studies were imported into Covidence™ (Melbourne, Australia). All results were independently reviewed by two investigators (RN, KW) for relevance based on titles and abstracts. Studies deemed relevant to the research question by both investigators were selected for full-text review. In cases of disagreement, a team discussion (HP, RN, KW) was conducted to reach a consensus.

All studies involving the use of VR, AR, and MR HMDs in preoperative educational discussions were included, provided they had a sample size of at least five participants. Exclusions were made for studies where patients did not use the HMDs (e.g. studies focused on surgical planning by surgeons) and for those addressing other forms of education (e.g. student or resident training). Additionally, duplicates, systematic reviews, meta-analyses, commentaries, and letters to the editor outside the scope of the topic were excluded. The inclusion process is illustrated in a flow diagram in Fig. 2.

Extracted data included evidence level, study details (author, publication date, clinic and country of study, number of patients, patient age and gender, study design with intervention and control groups), and the measured impact of the preoperative use of VR, AR, and MR HMDs on informed consent, patients’ education, patients’ satisfaction and patients’ anxiety. Interventions were categorized according to the type of visualization tool used: VR, AR, or MR.

### Level of Evidence and Quality Assessment

The risk of bias was assessed using the Methodological Index for Non-Randomized Studies (MINORS), which allows for the quality assessment of randomized, non-randomized, comparative, and non-comparative studies. For non-comparative studies, four domains are omitted [23]. We modified the MINORS tool by excluding two domains (D) that assess the follow-up period, as this aspect was irrelevant to our included study types, and none of the studies conducted a follow-up.

Two authors (RN, KW) independently assessed the included studies, with disagreements resolved through discussion (HP).

**Fig. 1:**
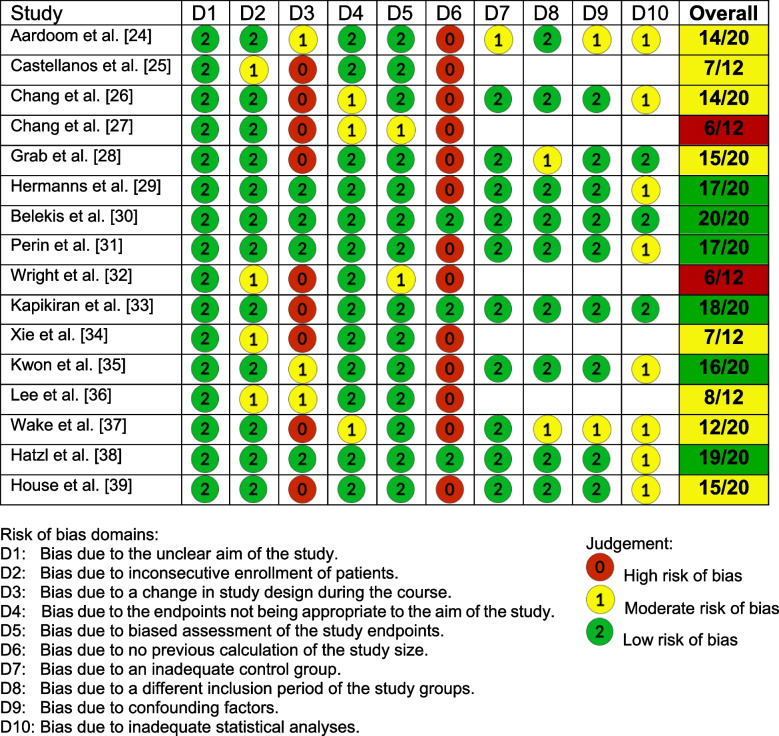
Risk of bias evaluation using the Methodological Index for Non-Randomized Studies (MINORS)

The maximum achievable score for each study type was divided by four, and studies were rated based on this scale. Non-comparative studies were categorized as very high risk (0–3), high risk (4–6), medium risk (7–9), and low risk (10–12). Comparative studies were categorized as very high risk (0–5), high risk (6–10), medium risk (11–15), and low risk (16–20). Among the included studies, 2 were found to have a high risk of bias, 8 showed a moderate risk of bias, and 6 demonstrated a low risk of bias (Fig. 1).

## Results

### Results of the Literature Search

**Fig. 2:**
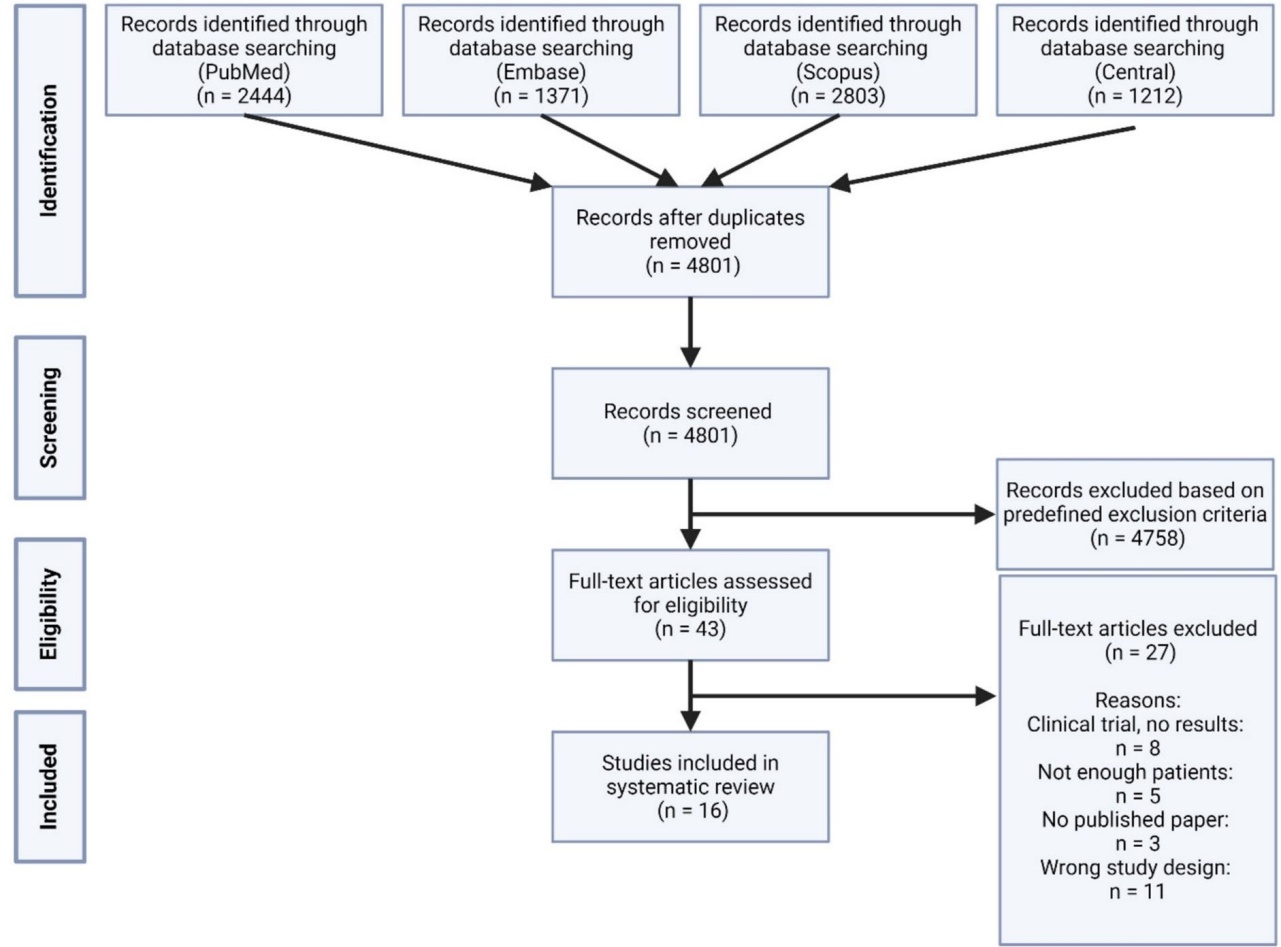
PRISMA flow chart depicting the different phases of the systematic review

The search yielded 7830 items, from which 3022 duplicates were removed. Of the remaining 4801 articles, 4758 were deemed irrelevant to the scope of this review. Consequently, 43 papers were selected for full-text review. Following the application of inclusion criteria, 16 papers were included in the final analysis (Fig. 2). The results were conducted using the PICO framework, which also provided the reasons for excluding studies after full-text screening.

### Study Characteristics

The included papers comprised the following study designs: Randomized Controlled Trials (RCTs) (n = 10) and Non-Randomized Controlled Trials (non-RCTs) (n = 6). The non-RCTs included one comparative study and five non-comparative studies. The study characteristics are summarized in Tables 2, 3, 4, 5, 6 and 7 in the appendix.

**Table 2:** Study characteristics (NRS = Non-randomized study; U = Understanding; S = Satisfaction; A = Anxiety; **↑** Increased; = No difference; **↓** Reduced)

Study	HMD	Study design	Specialty	*Participants*	Results
Aardoom et al. 2022, Netherlands [24]	VR	Non-RCT	Cardiology	N = 8	S: =
Castellanos et al. 2020, USA [25]	VR	NRS (non-comparative)	Cardiology	N = 46	U: ↑; S: =
Chang et al. 2021, Taiwan [26]	VR	RCT	Cardiology	N = 33	U: ↑; S: ↑; A ↓
Chang et al. 2021, Taiwan [27]	VR	NRS (non-comparative)	Cardiology	N = 32	U: ↑; S: =
Grab et al. 2023, Germany [28]	VR	RCT	Cardiology	N = 99	U: ↑; S: ↑; A ↓
Hermans et al. 2023, Netherlands [29]	VR	RCT	Cardiology	N = 134	S: ↑; A ↓
Bekelis et al. 2017, USA [30]	VR	RCT	Neurosurgery	N = 127	S: ↑; A ↓
Perin et al. 2021, Italy [31]	VR	RCT	Neurosurgery	N = 33	U: ↑; S: ↑; A =
Wright et al. 2021, USA [32]	VR	NRS (non-comparative)	Neurosurgery	N = 50	U: ↑; S: ↑
Kapikiran et al. 2022, Turkey [33]	VR	RCT	Organ transplant surgery	N = 120	S: ↑; A ↓
Xie et al. 2021, USA [34]	VR	NRS (non-comparative)	Organ transplant surgery	N = 14	U: ↑; A ↓
Kwon et al. 2023, Korea [35]	VR	RCT	Plastic and reconstructive surgery	N = 80	U: ↑; S: ↑; A ↓
Lee et al. 2022, USA [36]	AR	NRS (non-comparative)	Neurosurgery	N = 24	U: ↑; S: ↑
Wake et al. 2019, USA [37]	AR	RCT	Urology	N = 200	U: =; S: ↑
Hatzl et al. 2023, Germany [38]	MR	RCT	Vascular Surgery	N = 50	U: =; S: ↑
House et al. 2019, Germany [39]	MR	RCT	Neurosurgery	N = 17	U: ↑; S: ↑; A ↓

### Description of the Patient Population

A total of 1067 patients were included in this systematic review, with 32% being female and 68% being male. The low proportion of female participants is primarily due to the study by Wake et al. (10% women), which focused on prostate and kidney cancer, and studies by Hatzl et al. (12% women) and Grab et al. (13% women), which involved patients with vascular diseases [28, 37, 38].

The average age of patients from 14 of 16 studies is 57.4 years. Two studies reported patient age in ranges, excluding them from the average calculation. Castellanos et al. [25] reported 4% of patients aged 41–60 years and 96% over 61 years, while Chang et al. reported 6% aged 30–40 years, 15% aged 40–50 years, 24% aged 50–60 years, and 55% over 60 years [25, 26].

The systematic review includes studies from various specialties: cardiology (n = 6), neurosurgery (n = 5), organ transplant surgery (n = 2), vascular surgery (n = 1), plastic and reconstructive surgery (n = 1), and urology (n = 1) [24–39] (Table 2).

### Used Interventions

For the descriptive presentation of the results, the studies were divided into three categories based on the interventional visualization tool used: VR HMD (n = 12), AR HMD (n = 2), and MR HMD (n = 2) [24–39]. Details on the interventional visualization tools are presented in Tables 2, 3, 4, 5, 6, and 7.

### Description of the Study Outcomes

This systematic review focuses on the impact of using different HMDs on patients’ understanding, satisfaction, and anxiety.

The term"patients’ understanding"encompasses various terms used in the included studies, such as"patient knowledge", “informational gain”, “procedural knowledge”, “patients comprehension” and “information desire” [27, 28, 31, 35, 38, 39]. For simplicity, all these outcomes are summarized as"patients’ understanding".

Patients’ satisfaction was measured directly with questionnaires in some studies, while others used additional terms such as “usability”, “effectiveness”, “self-efficacy”, “preferred patient education tool”, “comfort level”, “usefulness”, “happiness” and “Patient-Doctor Relationship” [24–27, 29, 30, 32, 33, 35–39]. For simplicity, all these outcomes are summarized as"patients’ satisfaction."

Objective outcomes were assessed using pre- and post-intervention questionnaires, and post-intervention questionnaires only.

#### Patients’ Understanding

Among the 16 studies using VR, AR, or MR HMDs in preoperative informed consent, 12 examined patients’ understanding. Of these, 8 studies utilized VR HMDs, two studies used AR HMDs, and two studies used MR HMDs [25–28, 31, 32, 34–39].

All 8 studies employing VR HMDs reported positive effects on patients’ understanding. Four non-randomized, non-comparative studies indicated improved patients’ understanding, with Wright et al. demonstrating statistically significant improvement in patients undergoing elective craniotomy [32]. The other three studies reported an improved understanding with no information of statistical significance in 98% of participating patients with cardiac interventions, in anatomic understanding of kidney patients, and in 97% of participating patients about their upcoming atrial fibrillation ablation procedures [25, 27, 34].

Four RCTs evaluated the impact of VR HMDs compared to standard educational methods including 2D images, verbal education, and paper education [26, 28, 31, 35]. In the study by Grab et al., patients were divided into three groups: one receiving VR-HMD-based education about their cardiac surgery, another using 3D printed models, and a control group. The VR-HMD group demonstrated the most significant statistical improvement in understanding their medical condition after informed consent [28]. Perin et al. also included two intervention groups, one using VR-HMDs for brain tumor visualization and the other utilizing a 3D screen. Patients in the VR-HMD group exhibited the highest level of understanding, significantly surpassing the control group [31]. Kwon et al. found that patients educated with VR-HMDs had a significantly lower information desire regarding their plastic or reconstructive surgery compared to the control group, indicating a better understanding of their condition and the procedure [35]. Additionally, Chang et al. reported that patients undergoing catheter ablation for atrial fibrillation who received VR-HMD education showed improved pre-procedure knowledge compared to those in the control group [26].

Among the two studies that examined patients’ understanding using AR HMDs, the results differ. In the non-randomized non-comparative study by Lee et al., all patients reported a statistically significant improved understanding of their medical condition using AR HMDs, whereas Wake et al. found no improvement compared to 2D imaging used in the control group in their RCT [36, 37]. In the study by Wake et al., patients’ understanding was additionally examined by using 3D printed models and visualization as 3D computer models in further intervention groups. The use of 3D printed models significantly improved patients’ understanding. The 3D computer model also performed significantly better as an intervention group than the AR HMD compared to the control group [37].

**Fig. 3:**
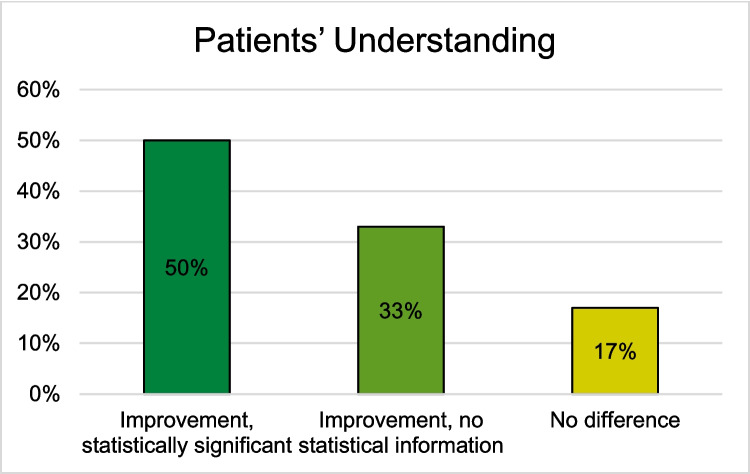
Patients’ understanding of their disease after the informed consent consultation using a VR, AR, or MR HMD compared to the control group (Improvement, statistically significant (N = 6): [28, 31, 32, 35, 36, 39]; Improvement, no statistical information (N = 4): [25–27, 34]; No difference (N = 2): [37, 38])

Studies utilizing MR HMDs generated mixed results. Notably, the variation in control group visualization methods between the two studies limits their comparability. In an RCT by Hatzl et al., the use of MR HMDs during preoperative informed consent was compared with 2D images displayed on a monitor. Both groups exhibited improved patients’ understanding compared to the pretest, but no significant difference was found between the two groups [38]. In contrast, House et al. compared MR HMDs with a rubber brain model, demonstrating that patients’ understanding was statistically significant higher when using the MR HMD compared to the rubber brain model (Fig. 3) [39]. Additional detailed information can be found in Tables 3, 4, 5, 6, 7, and 8 in the appendix.

#### Patients’ Satisfaction

Of the 16 studies that utilized VR, AR, or MR HMDs in preoperative informed consent, 15 examined patients’ satisfaction. The only study that did not assess this outcome was conducted by Xie et al. [34].

All 11 studies that employed VR HMDs reported positive effects on patients’ satisfaction. Three of these were non-randomized, non-comparative studies. Castellanos et al. [25] noted a 24.3% increase in patients’ satisfaction post-VR HMD use, while Chang et al. reported that 96% of patients were happy with the educational process facilitated by VR HMDs. Neither study provided information on statistical significance. In contrast, Wright et al. found a statistically significant improvement in the patient-physician relationship post VR-HMD education [25, 27, 32].

Aardoom et al. [24] conducted the only non-randomized comparative study, which evaluated patients’ satisfaction with VR HMD-informed consent both in the hospital and at home. Both settings yielded high satisfaction, with higher levels noted in the hospital [24].

Among the seven RCTs investigating VR HMDs, four compared them with paper-based education. Chang et al. and Bekelis et al. observed improved satisfaction without reporting the statistical significance [26, 30]. Grab et al. and Hermans et al. reported statistically significant improvements in patients’ satisfaction with VR HMDs compared to the control group [28, 29]. Grab et al. also noted that satisfaction with a 3D-printed heart model was intermediate between the control and VR HMD groups [28]. Kapikiran et al. and Kwon et al. used verbal education for control groups, both reporting higher patients’ satisfaction with VR HMDs; Kapikiran et al.’s results were statistically significant, while Kwon et al.’s were not [33, 35]. Perin et al. found high satisfaction in both intervention groups receiving tumor visualization using VR HMDs and a 3D screen, although the control groups’ satisfaction was not reported [31].

In studies using AR HMDs, Lee et al. found all patients satisfied in their non-randomized, non-comparative study [36]. Conversely, Wake et al. observed significantly greater satisfaction in the AR HMD group in their comparative study [37].

**Fig. 4:**
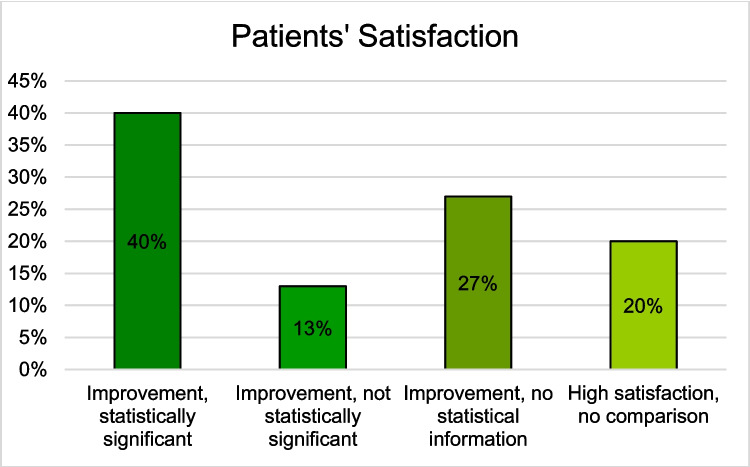
Patients’ satisfaction with the preoperative informed consent consultation using a VR, AR, or MR HMD compared to the control group (Improvement, statistically significant (N = 6): [28, 29, 32, 33, 37, 39]; Improvement, not statistically significant (N = 2): [35, 38]; Improvement, no statistical information (N = 4): [25–27, 30]; High satisfaction, no comparison (N = 3): [24, 31, 36])

Regarding MR HMDs, Hatzl et al. reported non-significantly higher satisfaction in the MR HMD group compared to the 2D image control group [38]. House et al. found significantly higher satisfaction in the intervention group compared to a rubber brain model control, by allowing patients to choose their preferred patient education tool (Fig. 4) [39]. Additional detailed information can be found in Tables 3, 4, 5, 6, 7, and 8 in the appendix.

#### Patients’ Anxiety

Overall, 9 out of 16 studies utilizing VR, AR, or MR HMDs in preoperative informed consent examined patients’ anxiety. Notably, none of the studies in the AR HMD category assessed patients’ anxiety.

**Fig. 5:**
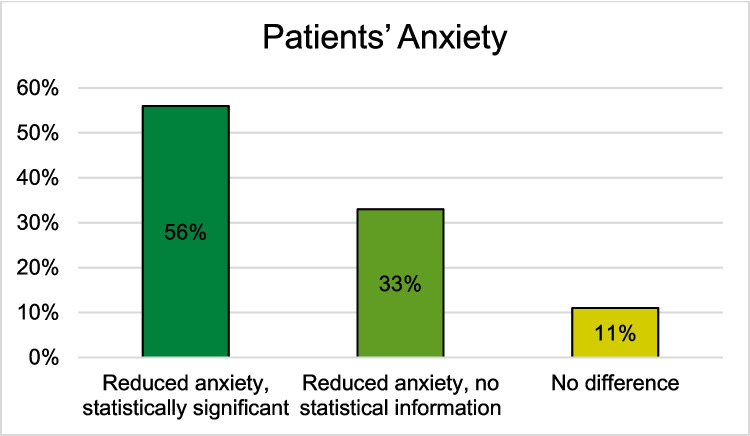
Patients’ preoperative anxiety after the informed consent consultation using a VR, AR, or MR HMD compared to the control group (Reduced anxiety, statistically significant (N = 5): [28, 29, 33, 35, 39]; Reduced anxiety, no statistical information (N = 3): [26, 30, 34]; No difference (N = 1): [31])

For VR HMDs, 7 out of 8 studies observed a reduction in anxiety following the preoperative educational discussion [26, 28–30, 33–35]. The RCT by Perin et al. was the only study reporting no difference in patients’ anxiety between the two intervention groups and the control group [31]. Hermans et al. present mixed results: while the proportion of patients with anxiety was statistically significantly lower in the VR HMD intervention group, the information and anxiety scores of both study groups were comparable [29]. Grab et al. and Kapikiran et al. reported statistically significant lower patients’ anxiety after informed consent using VR HMDs compared to before education [28, 33]. Kwon et al. found significantly lower patients’ anxiety in the VR HMD intervention group compared to the verbal control group [35]. Xie et al., Chang et al., and Bekelis et al. reported reduced anxiety in patients regarding their surgery after receiving informed consent via VR HMDs, although they did not provide information on statistical significance [26, 30, 34]. Among the studies using MR HMD for preoperative informed consent, House et al. reported a statistically significant reduction in patients’ anxiety as a result of the education process (Fig. 5) [39]. Additional detailed information can be found in Tables 3, 4, 5, 6, 7, and 8 in the appendix.

Overall,12 studies examined patients’ understanding, of which 50% reported a statistically significant improvement, 33% reported an improvement without any information about statistical significance, and 17% reported no difference using HMDs in the preoperative informed consent [25–28, 31, 32, 34–39] (Fig. 3). Regarding patients’ satisfaction, 33% of the 15 studies that examined patients’ satisfaction reported statistically significant improvement, 13% reported a non-significant improvement, 27% reported an improvement without any information about statistically significance, and 7% reported no difference using HMDs [24–26, 28–30, 32, 33, 35, 37–39]. Additionally, 20% reported high satisfaction without comparative value (Fig. 4) [27, 31, 36]. Concerning patients’ preoperative anxiety, 56% of the 9 studies reported a statistically significant reduction, 33% reported a reduction without any information about the statistical significance, and 7% reported no difference using HMDs in preoperative informed consent (Fig. 5) [26, 28–31, 33–35, 39].

## Discussion

This systematic review synthesizes current literature on the use of Virtual Reality (VR), Augmented Reality (AR), and Mixed Reality (MR) head-mounted displays (HMDs) in preoperative informed consent. It highlights the growing application and potential of these technologies in improving patients’ education, satisfaction, and reducing preoperative anxiety.

### Key Findings

Most included studies focused on VR HMDs, likely due to their earlier development and greater availability compared to AR and MR. The findings consistently indicate that these visualization tools enhance patient understanding and satisfaction, while also lowering anxiety. Many studies reported significant improvements in patients’ understanding and satisfaction, along with a notable reduction in preoperative anxiety [28, 29, 31–33, 35–37, 39]. Specifically, VR HMDs demonstrated the most consistent benefits; results for AR and MR were more heterogeneous.

### Comparison with Further Literature

While many studies have explored VR, AR, and MR in healthcare, there is a lack of comprehensive systematic reviews focusing on the use of these HMDs in multidisciplinary preoperative patient education. This gap is likely due to the recent adoption of these technologies, particularly MR, in the medical field. Prior reviews often focus on specific specialties or intraoperative use, rather than on informed consent [11, 12, 40–44]. The key findings of this review are further supported by studies that, despite not meeting the inclusion criteria, still demonstrated that HMDs can improve patients’ understanding and communication in surgical contexts [10, 45–47].

### Clinical Implications

Integrating VR, AR, and MR HMDs into preoperative education aligns with patient-centered care by offering immersive, interactive tools that clarify complex medical information. These technologies promote informed decision-making and can strengthen patient autonomy and satisfaction. The anxiety-reducing effects further suggest positive implications for surgical outcomes. In complex or high-risk cases, their ability to facilitate shared decision-making is particularly valuable. Clinicians should consider implementing these tools to improve the quality and clarity of preoperative consultations.

### Bias Risk and Study Outcomes

An important aspect of the review was the assessment of bias risk, using the MINORS tool. Upon analysis, we found no consistent correlation between the risk of bias and the statistical significance of the study outcomes. Both studies with high risk of bias [27, 32] and those with low risk of bias [29–31, 33, 35, 38] reported significant findings. However, studies with a lower risk of bias, reflecting higher methodological quality, are generally more reliable and provide stronger evidence for the reported outcomes.

### Limitations

Included studies varied in methodology, sample size, and study design. Research on AR and MR often involved smaller samples, limiting generalizability [36, 39]. While most studies employed rigorous methodologies, including randomized controlled trials (RCTs), some non-comparative studies and one non-randomized comparative study were also included [26, 28–31, 33, 35, 37–39].

A significant limitation identified was the variability in the amount of pre-information provided to patients across studies [29, 34, 37]. This heterogeneity complicates the comparison of outcomes, as the baseline level of patient knowledge before the HMD-based informed consent varied. Additionally, the use of varied and sometimes unvalidated assessment tools complicates data synthesis [27–39]. Future research should aim for standardised pre-information and validated outcome measures to improve reliability.

### Future Research

Future research on AR and MR HMDs should involve larger and more diverse patient populations to improve the generalizability of findings. Additionally, comprehensive evaluations of cost-effectiveness are crucial to support broader clinical adoption, taking into account not only implementation costs but also potential savings from reduced anxiety and improved outcomes. Investigating long-term impacts on surgical success and patient satisfaction remains equally important.

## Conclusion

This systematic review provides evidence supporting the use of VR, AR, and MR HMDs in preoperative education and informed consent processes. These technologies offer a promising approach to enhancing patients’ understanding, satisfaction, and emotional well-being in the perioperative period. By leveraging HMD-based interventions, healthcare providers can foster more meaningful patient engagement and facilitate shared decision-making, ultimately improving the quality of care delivered to surgical patients. The integration of these advanced visualization tools into clinical practice represents a significant step forward in patient education and engagement.

It should be noted, however, that most of the available evidence relates to the use of VR, as fewer studies have reported results for AR and MR. As the technology continues to evolve, further research and standardisation of methodologies will be essential to fully realize the potential of VR, AR, and MR HMDs in enhancing preoperative care.

## Data Availability

No datasets were generated or analysed during the current study.

## Appendix

Lists of abbreviations

**Table:**

Abbreviation	Meaning
AR	Augmented Reality
CT	Computerized Tomography
D	Domain
HMD	Head-mounted display
HMDs	Head-mounted displays
MINORS	Methodological Index for Non-Randomized Studies
MR	Mixed Reality
NRS	Non-Randomized Study
Non-RCT	Non-Randomized Controlled Trial
RCT	Randomized Controlled Trial
VR	Virtual Reality

### Search Strategy

**Pubmed:**

(virtual reality[Mesh] OR"Virtual Realit*"[Tiab] OR Mixed Reality[MESH] OR"Mixed Realit*"[Tiab] OR Augmented Reality[Mesh] OR"Augmented Realit*"[Tiab] OR holography[Mesh] OR"three-dimensional hologram"[Tiab] OR"three-dimensional virtual model"[Tiab] OR"3D holograms"[Tiab] OR"surgical visualization"[Tiab] OR"holograms"[Tiab] OR"preoperative imaging"[Tiab] OR"preoperative planning"[Tiab] OR"holographic"[Tiab] OR"3D visualization"[Tiab] OR"3D reconstruction"[Tiab] OR"Surgery, Computer-Assisted"[Mesh]).

AND

("surgery"[Subheading] OR"surgical"[Tiab] OR"surgery"[Tiab] OR"operative"[Tiab] OR"operation"[Tiab]).

AND

("Informed Consent"[Mesh] or"Informed Consent"[Tiab] OR"Patient Education as Topic"[Mesh] OR"Patient Participation"[Mesh] OR"Patient Participation"[Tiab] OR"Patient Education"[Tiab] OR"preoperative communication"[Tiab] OR"medical comprehension"[Tiab] OR"objective comprehension"[Tiab] OR"Patient-physician relationship"[Tiab] OR"Preoperative Care"[Mesh] OR"intuitive communication"[Tiab]).

**Embase:**

(virtual reality.mp OR Virtual Realit*.ab,kf,ti OR Mixed Reality.mp OR Mixed Realit*.ab,kf,ti OR Augmented Reality.mp OR Augmented Realit*.ab,kf,ti OR holography.mp OR three-dimensional hologram.ab,kf,ti OR three-dimensional virtual model.ab,kf,ti OR 3D holograms.ab,kf,ti OR surgical visualization.ab,kf,ti OR holograms.ab,kf,ti OR preoperative imaging.ab,kf,ti OR preoperative planning.ab,kf,ti OR holographic.ab,kf,ti OR 3D visualization.ab,kf,ti OR 3D reconstruction.ab,kf,ti OR Surgery, Computer-Assisted.mp).

AND

(surgery.mp OR surgical.ab,kf,ti OR surgery.ab,kf,ti OR operative.ab,kf,ti OR operation.ab,kf,ti).

AND

(Informed Consent.mp OR Informed Consent.ab,kf,ti OR Patient Education as Topic.mp OR Patient Participation.mp OR Patient Participation.ab,kf,ti OR Patient Education.ab,kf,ti OR preoperative communication.ab,kf,ti OR medical comprehension.ab,kf,ti OR objective comprehension.ab,kf,ti OR Patient-physician relationship.ab,kf,ti OR Preoperative Care.mp OR intuitive communication.ab,kf,ti).

**Scopus:**

TITLE-ABS-KEY ("virtual reality"OR"Virtual Realit*"OR"Mixed Reality"OR"Mixed Realit*"OR"Augmented Reality"OR"Augmented Realit*"OR"holography"OR"three-dimensional hologram"OR"three-dimensional virtual model"OR"3D holograms"OR"surgical visualization"OR"holograms"OR"preoperative imaging"OR"preoperative planning"OR"holographic"OR"3D visualization"OR"3D reconstruction"OR"Surgery, Computer-Assisted").

AND

TITLE-ABS-KEY ("surgery"OR"surgical"OR"surgery"OR"operative"OR"operation").

AND

TITLE-ABS-KEY ("Informed Consent"OR"Informed Consent"OR"Patient Education as Topic"OR"Patient Participation"OR"Patient Participation"OR"Patient Education"OR"preoperative communication"OR"medical comprehension"OR"objective comprehension"OR"Patient-physician relationship"OR"Preoperative Care"OR"intuitive communication").

**Central:**

(MeSH descriptor: [virtual reality] OR"Virtual Realit*":ti,ab,kw OR MeSH descriptor: [Mixed Reality] OR"Mixed Realit*":ti,ab,kw OR MeSH descriptor: [Augmented Reality] OR"Augmented Realit*":ti,ab,kw OR MeSH descriptor: [holography] OR"three-dimensional hologram":ti,ab,kw OR"three-dimensional virtual model":ti,ab,kw OR"3D holograms":ti,ab,kw OR"surgical visualization":ti,ab,kw OR"holograms":ti,ab,kw OR"preoperative imaging":ti,ab,kw OR"preoperative planning":ti,ab,kw OR"holographic":ti,ab,kw OR"3D visualization":ti,ab,kw OR"3D reconstruction":ti,ab,kw OR MeSH descriptor: [Surgery, Computer-Assisted]).

AND

(MeSH descriptor: [General Surgery] OR"surgical":ti,ab,kw OR"surgery":ti,ab,kw OR"operative":ti,ab,kw OR"operation":ti,ab,kw).

AND

(MeSH descriptor: [Informed Consent] OR"Informed Consent":ti,ab,kw OR MeSH descriptor: [Patient Education as Topic] OR MeSH descriptor: [Patient Participation] OR"Patient Participation":ti,ab,kw OR"Patient Education":ti,ab,kw OR"preoperative communication":ti,ab,kw OR"medical comprehension":ti,ab,kw OR"objective comprehension":ti,ab,kw OR"Patient-physician relationship":ti,ab,kw OR MeSH descriptor: [Preoperative Care] OR"intuitive communication":ti,ab,kw).

### Study Overview

Tables 3, 4, 5, 6, 7, and 8.

**Table 3:** Virtual Reality Study Information

Author (year), Country	Interventional visualization tool	Study design	Specialty	Population characteristics	Outcome
Aardoom et al. 2022, Netherlands [24]	VR, Oculus Rift Go	Non-randomized comparative study	Cardiology	N = 8 patients with cardiac catheterization; mean age 67 years; 25% female	Patients’ experience and satisfaction
Castellanos et al. 2020, USA [25]	VR, Oculus	Non-randomized, non-comparative study	Cardiology	N = 46 patients with transcatheter aortic valve replacement (TAVR) and left atrial appendage occlusion (LAAO); 96% > 61 years; 39% female	Patients’ satisfaction and understanding
Chang et al. 2021, Taiwan [26]	VR HMD, no further information	Randomized controlled trial, comparative study	Cardiology	N = 33 patients with catheter ablation of atrial fibrillation; 54.5% > 60 years; female 51.5%	Patients’ satisfaction, self-efficacy (Anxiety, familiarity, and confidence), knowledge
Chang et al. 2021, Taiwan [27]	VR HMD, no further information	Non-randomized, non-comparative study	Cardiology	N = 32 patients with catheter ablation of atrial fibrillation	Patients’ satisfaction and knowledge
Grab et al. 2023, Germany [28]	VR, Oculus Quest 2	Randomized controlled trial, comparative study	Cardiology	N = 99 Patients with coronary artery bypass graft, surgical aortic valve replacement, and thoracic aortic aneurysm surgery; mean age 64.8 years; 13% female	Patients’ satisfaction, understanding, and presurgical anxiety
Hermans et al. 2023, Netherlands [29]	VR, Oculus Rift Go	Randomized controlled trial, comparative study	Cardiology	N = 134 patients with atrial fibrillation ablation; mean age 66; 38.1% female	Patients’ anxiety and satisfaction
Bekelis et al. 2017, USA [30]	VR, Oculus	Randomized controlled trial, comparative study	Neurosurgery	N = 127 patients with cranial and spinal operations; mean age 55.3 years; 41.9% female	Patients’ satisfaction and experience
Perin et al. 2021, Italy [31]	VR, Oculus Rift	Randomized controlled trial, comparative study	Neurosurgery	N = 33 patients with brain tumor surgery; mean age 49.4 years; 42.4% female	Patients’ understanding, anxiety, and satisfaction
Wright et al. 2021, USA [32]	VR, Oculus Rift	Non-randomized, non-comparative study	Neurosurgery	N = 50 patients with elective craniotomy for aneurysm or tumor resection; mean age 57.5 years; 48% female	Patients’ understanding and Patient-Doctor Relationship
Kapikiran et al. 2022, Turkey [33]	VR HMD, no further information	Randomized controlled trial, comparative study	Organ transplant surgery	N = 120 patients with liver transplant surgery; mean age 50.5 years; 28.3% female	Patients’ satisfaction and anxiety
Xie et al. 2021, USA [34]	VR, Oculus Rift	Non-randomized, non-comparative study	Organ transplant surgery	N = 14 patients with laparoscopic donor nephrectomy; mean age 42; 60% female	Patients’ understanding and anxiety
Kwon et al. 2023, Korea [35]	VR, PICO G2	Randomized controlled trial, comparative study	Plastic and reconstructive surgery	N = 80 patients with plastic or reconstructive surgery; mean age 41.8; 35% female	Patients’ satisfaction, anxiety, and information desire

**Table 4:** Virtual Reality Study Results

Author (year), Country	Intervention	Control	Questionnaire	Results	Quality of study
Aardoom et al. 2022, Netherlands[24]	VR interactive representation of the whole care process at the hospital	VR interactive representation of the whole care process at home	1: Client Satisfaction Questionnaire-8 (CSQ-8); 2: System Usability Scale (SUS); 3: Perceived Effectiveness Questionnaire	**Understanding:**/ **Satisfaction:** VR in hospital: CSQ-8: 29; SUS: 80 VR at home: CSQ-8: 25; SUS: 98 Perceived Effectiveness: All patients agreed that VR HMDs helped them feel more informed about the care process of the cardiac catheterization procedure **Anxiety:**/	**MINORS:** 14/20
Castellanos et al. 2020, USA [25]	VR virtual tour of the patient´s anatomy	No control	1: Online questionnaire regarding their 360°VR and prior consultations	**Understanding:** Before VR: 7/10 (SD 2,28), after VR: 9,24/10 (SD 1,28); VR improved patient **understanding** (98%) and **comfort level** (93%) with the treatment course Value of visualization of pathology and treatment by VR HMD: 9,43/10 (SD 1,49) **Satisfaction:** Before VR: 78%, After VR 97% **Anxiety:**/	**MINORS:** 7/12
Chang et al. 2021, Taiwan [26]	VR education	Paper education	1: Satisfaction questionnaire 2: Knowledge written test 3: Questionnaire for Patients’ self-assessed self-efficacy and satisfaction	**Understanding:** VR HMDs improved the pre-procedure knowledge of patients **Satisfaction:** VR HMDs increased the patient’s self-efficacy about the procedure, post-procedure self-care, and post-procedure self-monitoring-related knowledge The results indicate that the overall satisfaction of patients in the VR group with preoperative education and materials was higher than that of the patients in the control group **Anxiety:** VR HMDs decreased the in-procedure anxiety of VR group patients	**MINORS:** 14/20
Chang et al. 2021, Taiwan [27]	VR education	No control	1: Self-assessed questionnaire for Patients’ self-efficacy and satisfaction	**Understanding:** 90% of patients agreed that the use of VR HMDs on atrial fibrillation ablation met their needs 97% of patients agreed that the use of HMDs accurately provided knowledge of the atrial fibrillation ablation procedure **Satisfaction:** 96% of patients agreed that they were happy to have used the VR HMDs to increase their knowledge of the atrial fibrillation ablation procedure **Anxiety:**/	**MINORS:** 6/12
Grab et al. 2023, Germany [28]	VR model education, 3D-printed model education	Standardised pre-printed paper-based education models	1: Self-developed questionnaire to assess understanding and satisfaction 2: German short version of the State-Trait-Anxiety-Inventory (STAI) 3: Visual Analog Scale (VAS) to assess anxiety	**Understanding:** Procedural knowledge revealed a highly statistically significant increase in each group after patient education (control group: 65.44% to 80.36%, p < 0.0001, 3D model: 68.17% to 83.46%, p < 0.0001, VR HMD: 67.62% to 87.98%, p < 0.0001) Patient understanding of the surgical procedure showed better results with VR (control: 84.60 ± 8.26% and VR HMD: 92.42 ± 8.15%, p = 0.011), as well as a better visualization (control: 86.10 ± 11.53% and VR HMD: 93.55 ± 9.15%, p < 0.017) **Satisfaction:** Patients were more satisfied using VR HMDs rather than paper sheets (control: 84.80 ± 12.74%, VR: 93.33 ± 9.58%, p < 0.0038) Patients rated the quality of patient education using both visualization methods (3D and VR models) higher compared to conventional paper-based methods (control: 86.32 ± 11.89%, 3D: 94.12 ± 9.25%, p < 0.0095, VR: 92.90 ± 11.01%, p < 0.0412) **Anxiety:** Results showed a significant decrease in VAS anxiety in patients educated with VR HMDs (5.00 to 4.32, p < 0.0001) and a lower, but not statistically significant, STAI (24.32 to 23.00)	**MINORS:** 15/20
Hermans et al. 2023, Netherlands [29]	Standard preprocedural information through oral counseling, information leaflets, and short dedicated 360° VR video	Standard preprocedural information through oral counseling and information leaflets	1: Amsterdam Preoperative Anxiety and Information Scale (APAIS) together with some additional questions concerning procedural experience, ease of use of the disposable cardboard VR viewer and in-hospital VR headset, and satisfaction with both pre-and post-ablation	**Understanding:**/ **Satisfaction:** After ablation, more patients who had applied a VR HMD were satisfied with the preprocedural information than before ablation (post-ablation n = 40 (83.3%) vs. pre-ablation n = 29 (60.4%), P = 0.007) **Anxiety:** Fewer patients in the VR group reported worries about the ablation procedure than in the control group (VR HMD: n = 13 (19.1%) and control group: n = 27 (40.9%), P = 0.006). Information and anxiety scores were comparable between VR and the control group	**MINORS:** 17/20
Bekelis et al. 2017, USA [30]	5-min VR video describing the preoperative and postoperative experience for the day of the surgery	Standard preoperative experiences were provided with routine audiovisual descriptions of the preoperative experience	1: Evaluation du Vecu de l’Anesthesie Generale (EVAN-G) score 2: Amsterdam Preoperative Anxiety and Information (APAIS) score 3: VAS for pain, satisfaction, stress, and preparedness	**Understanding:**/ **Satisfaction:** Patients who used VR HMDs reported higher satisfaction: EVAN-G: Control group: 64.3 (SD, 11.7), VR group: 84.3 (SD, 6,4), APAIS: Control group: 60,8, VR group: 90,7. The use of VR HMDs resulted in higher VAS satisfaction scores (difference, 33.2; 95% CI, 25.4–41.0) and a higher postoperative VAS satisfaction score (difference, 26.4; 95% CI, 20.1–32.6) **Anxiety:** The use of VR HMDs led to a lower average preoperative VAS stress score (difference: –41.7; 95% CI, –33.1 to –50.2) and to a higher preoperative VAS preparedness (difference: 32.4; 95% CI, 24.9–39.8)	**MINORS:** 20/20
Perin et al. 2021, Italy [31]	3D planner Surgical Theater™ (VR) and 3D planner Vesalius™ (3D glasses in front of a 3D screen)	Standard MR and/or CT 2D DICOM images	1: Spielberger State and Trait Anxiety Self-evaluation Questionnaire (State Anxiety Inventory (STAI Y-1) and Trait Anxiety Inventory (STAI Y-2)) to assess anxiety 2: Ad hoc Knowledge-Comprehension Questionnaire	**Understanding:** Interventional groups reached greater comprehension: group 1 (VR): 82.65 (6.83), group 2 77.76 (10.19), and control group 57.70 (12.49) (P < 0.001; F = 18.83. Subjective comprehension did not differ between groups: group 1: 88.33 (13.17), group 2: 89.76 (11.63), and control group: 89.09 (17.00), P = 0.97; F = 0.027. Objective comprehension resulted in a significant difference between groups: group 1 (VR) 92.73 (10.09), group 2: 92.73 (10.09) and control group: 67.57 (27.41) (group 1 vs. control: P = 0.007; group 2 vs. control: P = 0.007; F = 7.289). Experimental groups reported a greater comprehension of possible complications compared to the control group (group 1 (VR): 55.83 (24.21), group 2: 48.37 (11.15) and control group: 28.22 (16.73)) (group 1 vs. control group P = 0.004; group 2 vs. control P = 0.034; F = 6.768) **Satisfaction:** Patients rated the usefulness of these devices in understanding their pathology with a mean (SD) of 4.73 (0.65) in group 1 (VR) and 4.73 (0.65) in group 2 **Anxiety:** No statistically significant differences between the 3 groups in anxiety levels: STAI Y-1 group 1: 54.10 (10.25), group 2: 55.20 (13.12), and control group: 56.45 (9.23)	**MINORS:** 17/20
Wright et al. 2021, USA [32]	VR HMD displaying surgical anatomy and pathology or Transportable touch-screen VR monitor displaying surgical anatomy and pathology	No control	1: Modified PatientDoctor Relationship Questionnaire (PDRQ-9)	**Understanding and Satisfaction:** The mean total PDRQ-9 score after consent was significantly higher than the mean score before consent (post: 50.53 vs. pre: 45.79, p = 0.001). PDRQ-9 includes questions about satisfaction and understanding **Anxiety:**/	**MINORS:** 6/12
Kapikiran et al. 2022, Turkey [33]	VR HMD showing preoperative 34 min training video	Verbal preoperative training	1: Patient information form developed by the researchers 2: Anxiety specific to surgery questionnaire (ASSQ) 3: Newcastle Satisfaction with Nursing Care Scale (SNCS)	**Understanding:**/ **Satisfaction:** The SNCS score revealed a statistically significant difference between the pre-and post-test mean SNCS values in the VR-HMD group (66.6 vs. 79.4, p = 0.000). No statistically significant difference was found between the pretest and posttest mean SNCS values of patients in the control group (66.5 vs. 68.9, p = 0.516) **Anxiety:** The difference between the mean pretest and posttest ASSQ scores of the experiment group (VR) was statistically significant (37.6 vs. 29.0, p = 0.001). No statistically significant difference was found between the mean pretest and posttest ASSQ scores of the patients in the control group (37.6 vs. 36.66 p = 0.807). Paired–samples t-test revealed a significant relationship between the participants’ mean ASSQ and SNCS scores, and this relationship was found to be moderately negative (r = -0.613, p =.000)	**MINORS:** 18/20
Xie et al. 2021, USA [34]	CT visualization of patient´s anatomy, afterward VR visualization	No control	1: Interactive Virtual Reality (iVR) surgeon and patient questionnaires	**Understanding:** Patients agreed that the iVR model gave them a better understanding of the size and shape of their kidneys (5/5) **Satisfaction:**/ **Anxiety:** Before education, patients were somewhat concerned about living donor nephrectomy surgery (3/5). However, patients indicated that they were less concerned about their surgery after viewing the iVR model (4.5/5)	**MINORS:** 7/12
Kwon et al. 2023, Korea [35]	VR surgery experience education	Conventional verbal education	1: Amsterdam Preoperative Anxiety Information Scale (APAIS) 2: In-hospital patient satisfaction questionnaire	**Understanding:** APAIS-I scores for information desire after preoperative education were 4.33 ± 0.97 in the VR group and 6.93 ± 1.31 in the control group, indicating that there is a statistically significant difference between the two groups (t = 10.09, p < 0.001) **Satisfaction:** The satisfaction survey scored 28.35 ± 4.92 in the VR group and 26.30 ± 7.35 in the control group, indicating greater satisfaction in the VR group. However, the satisfaction of the two groups was not statistically significant (t = − 1.47, p = 0.147) **Anxiety:** The APAIS-A scores after preoperative education were 7.73 ± 1.52 in the VR group and 13.00 ± 1.16 in the control group, indicating a statistically significant difference between the two groups (t = 17.49, p < 0.001)	**MINORS:** 16/20

**Table 5:** Augmented Reality Study Information

Author (year), Country	Interventional visualization tool	Study design	Specialty	Population characteristics	Outcome
Lee et al. 2022, USA [36]	AR, visualization platform (ARVP; Surgical Theater, Inc) Headset Magic Leap	Non-randomized, non-comparative study	Neurosurgery	N = 24 patients with an identifiable pathology on intracranial imaging that required a neurosurgical consultation; mean age 53 years; 79.2% female	Patients’ understanding, satisfaction, and comfort level
Wake et al. 2019, USA [37]	AR, Microsoft HoloLens	Randomized controlled trial, comparative study	Urology	N = 200 patients with kidney or prostate cancer; mean age 63,6; 10% females	Patients’ understanding and comfort level

**Table 6:** Augmented Reality Study Results

Author (year), Country	Intervention	Control	Questionnaire	Results	Quality of study
Lee et al. 2022, USA [36]	Patients were allowed to use the 360-degree ARVP concurrently with the neurosurgeon. Further explanation of the patient´s pathology and treatment plan were discussed with the patient in the AR environment	No control	1: Patient Survey of 6 questions	**Understanding:** All patients (19 strongly agreed and 5 agreed) reported that using the 360 ARVP system helped them improve their understanding of their medical condition. Understanding rating before experiencing 360 ARVP mean rating was 6.8, after experiencing 360 ARVP mean rating was 9.3. The difference in mean score between patients’ understanding before and after VR HMD use was statistically significant (p < 0.0017) **Satisfaction:** All patients (20 strongly agreed and 4 agreed) reported that they were satisfied with their 360 ARVP experience. All but one patient either agreed or strongly agreed that the 360 ARVP experience helped them to feel more comfortable with their proposed treatment options and to feel involved in decisions about their treatment **Anxiety:**/	**MINORS:** 8/12
Wake et al. 2019, USA [37]	Pre-operative planning with imaging plus a patient-specific 3D model which was either 3D printed, visualized in AR, or viewed in a 3D computer model	Pre-operative planning with imaging alone	1: Likert-scale survey to assess patient understanding of disease and procedure 2: Survey to assess patient-perceived usefulness of 3D models	**Understanding and Satisfaction:** AR model showed no improvement over standard imaging in terms of understanding, but it did lead to a significant increase in user satisfaction. Patients had a better understanding of their anatomy and disease and felt more comfortable using 3D-printed models than AR models (range 4.60–4.70/5 vs. 3.50–4.23/5, p < 0.05) Patients perceived the 3D printed models to be more helpful than the AR models in terms of their understanding of the anatomy (9.21 ± 1.49 vs. 7.92 ± 2.84, p = 0.04). In addition, patients found the 3D printed models more valuable than the AR and 3D computer models in terms of their understanding of disease (9.11 ± 1.86 vs. 7.50 ± 3.35 vs. 8.59 ± 2.05, p < 0.05) **Anxiety:**/	**MINORS:** 12/20

**Table 7:** Mixed Reality Study Information

Author (year), Country	Interventional visualization tool	Study design	Specialty	Population characteristics	Outcome
Hatzl et al. 2023, Germany [38]	MR, Mixed Reality Viewer (BrainLab AG)	Randomized controlled trial, comparative study	Vascular Surgery	N = 50 patients with open or endovascular repair for juxtarenal or infrarenal abdominal aortic aneurysms; mean age 68.5 years; 12% female	Patients’ informational gain and satisfaction
House et al. 2019, Germany [39]	MR, HoloLens (Microsoft)	Randomized controlled trial, comparative study	Neurosurgery	N = 17 patients with epilepsy surgery and stereotactic implantation of deep brain stimulation (DBS) or stereo-EEG electrodes; mean age 36.1 years; 58.8% female	Patients’ informational gain, anxiety

**Table 8:** Mixed Reality Study Result

Author (year), Country	Intervention	Control	Questionnaire	Results	Quality of study
Hatzl et al. 2023, Germany [38]	Virtual, three-dimensional model of a patient-individual reconstruction of the vascular anatomy through the HMD (Mixed Reality Viewer, Brainlab AG, Munich, Germany)	Two-dimensional monitor using conventional viewing software	1: Informational Gain Questionnaire (IGQ) 2: Patient Satisfaction Questionnaire (PSQ) 3: MR Usability Questionnaire (MRUQ) (only Intervention Group)	**Understanding:** The MR group scored 6.5 (± 1.8) points on the informational gain questionnaire before patient education and 7.9 (± 1.5) points after patient education. The control group scored 6.2 (± 1.8) and 7.6 (± 1.6) points. 22 of 25 patients (88%) improved their score in the MR group while in the control group, 23 of 25 patients (92%) improved. Both groups showed a significant increase in the achieved score in IGQ (p < 0.01). However, there was no significant difference in mean improvement between the two groups with a mean difference in score of 1.4 points (± 1.8) in each group (p = 0.5) In the subjective evaluation questionnaire, 92% of patients indicated that they agreed or strongly agreed that using HMDs helped them to understand the disease **Satisfaction:** The mean patient satisfaction in the MR group was 18.3 (± 3.7) out of a maximum of 21 points. The control group scored 17 (± 3.6) points. There was no statistically significant difference in the results of the patient satisfaction questionnaire (p = 0.1) **Anxiety:/**	**MINORS:** 19/20
House et al. 2019, Germany [39]	General information on the upcoming surgery in the usual way of the informing doctor followed by additional consecutive education with MR HMDs	General information on the upcoming surgery in the usual way of the informing doctor followed by additional consecutive education with rubber brain model	1: Questionnaire (12 questions) about the experience with the MR HMD or rubber brain model	**Understanding:** Patients perceived their patient education to be highly significantly more understandable (r = 0.84, p = 0.001) and almost significantly more imaginable (r = 0.57, p = 0.020) when their physician used VSI patient education compared with the rubber model **Satisfaction:** Significantly more patients chose MR as their preferred patient education tool (r = 0.91, p < 0.001), and almost significantly more patients chose MR as their future standard patient education tool (p = 0.020, r = 0.56) **Anxiety:** Patients felt significantly less anxious as a result of MR education (r = 0.64, p = 0.008)	**MINORS:** 15/20

## Declarations

### Registration Details

Clinical trial number: not applicable.

## References

[1] Institute NC: Dictionary of Cancer. https://www.cancer.gov/publications/dictionaries/cancer-terms/def/informed-consent. Accessed 21 Mar 2023

[2] Shay LA, Lafata JE. Where is the evidence? A systematic review of shared decision making and patient outcomes. Med Decis Making. 2015;35(1):114-31. 10.1177/0272989x14551638. 25351843 10.1177/0272989X14551638 PMC4270851

[3] Say RE, Thomson R. The importance of patient preferences in treatment decisions--challenges for doctors. Bmj. 2003;327(7414):542-5. 10.1136/bmj.327.7414.542. 12958116 10.1136/bmj.327.7414.542 PMC192849

[4] Coulter A. When should you involve patients in treatment decisions? Br J Gen Pract. 2007;57(543):771-2. 17925131 PMC2151806

[5] Amarasekera SS, Lander RO. Understanding of informed consent and surgeon liability by the public and patients. J Orthop Surg (Hong Kong). 2008;16(2):141-5. 10.1177/230949900801600202. 18725660 10.1177/230949900801600202

[6] Milgram P, Takemura H, Utsumi A, Kishino F. Augmented reality: A class of displays on the reality-virtuality continuum. Telemanipulator and Telepresence Technologies. 1994;2351. 10.1117/12.197321.

[7] Rokhsaritalemi S, Sadeghi-Niaraki A, Choi S-M. A Review on Mixed Reality: Current Trends, Challenges and Prospects. Applied Sciences. 2020;10:636. 10.3390/app10020636.

[8] Speicher M, Hall BD, Nebeling M. What is mixed reality? *Proc. CHI. Conf. Hum. Factors Comput. Syst.* 1–5, 2019. 10.1145/3290605.3300767

[9] Brigham TJ. Reality Check: Basics of Augmented, Virtual, and Mixed Reality. Med Ref Serv Q. 2017;36(2):171-8. 10.1080/02763869.2017.1293987. 28453428 10.1080/02763869.2017.1293987

[10] Lu L, Wang H, Liu P, Liu R, Zhang J, Xie Y, et al. Applications of Mixed Reality Technology in Orthopedics Surgery: A Pilot Study. Front Bioeng Biotechnol. 2022;10:740507. 10.3389/fbioe.2022.740507. 35273954 10.3389/fbioe.2022.740507 PMC8902164

[11] Dubron K, Verbist M, Jacobs R, Olszewski R, Shaheen E, Willaert R. Augmented and Virtual Reality for Preoperative Trauma Planning, Focusing on Orbital Reconstructions: A Systematic Review. Journal of Clinical Medicine. 2023 10.3390/jcm12165203 10.3390/jcm12165203 PMC10455745 37629251

[12] Bui T, Ruiz-Cardozo MA, Dave HS, Barot K, Kann MR, Joseph K, et al. Virtual, Augmented, and Mixed Reality Applications for Surgical Rehearsal, Operative Execution, and Patient Education in Spine Surgery: A Scoping Review. Medicina (Kaunas). 2024;60(2). 10.3390/medicina60020332. 10.3390/medicina60020332 PMC10890632 38399619

[13] Barteit S, Lanfermann L, Bärnighausen T, Neuhann F, Beiersmann C. Augmented, Mixed, and Virtual Reality-Based Head-Mounted Devices for Medical Education: Systematic Review. JMIR Serious Games. 2021;9(3):e29080. 10.2196/29080. 34255668 10.2196/29080 PMC8299342

[14] Victores AJ, Huynh N, Butler EB, Takashima M. Three-dimensional imaging for sinus surgery informed consent. Otolaryngology - Head and Neck Surgery (United States). 2012;147(SUPPL. 2):P118. 10.1177/0194599812451438a262.

[15] Liang Y, Qiu L, Lu T, et al. OralViewer: 3D demonstration of dental surgeries for patient education with oral cavity reconstruction from a 2D panoramic X-ray. In: Proceedings of the ACM Conference. New York, NY, USA. New York: ACM, 553–63, 2021. 10.1145/3397481.3450695

[16] Hermann M. 3-dimensional computer animation--a new medium for supporting patient education before surgery. Acceptance and assessment of patients based on a prospective randomized study--picture versus text. Der Chirurg; Zeitschrift fur alle Gebiete der operativen Medizen. 2002;73(5):500‐7. 10.1007/s00104-001-0416-y. 10.1007/s00104-001-0416-y 12089836

[17] Wucherer P, Bichlmeier C, Eder M, et al. Multimodal medical consultation for improved patient education. In: *Proceedings of the Conference on Image Processing for Medicine 2010 (BVM 2010)*. Aachen: CEUR Workshop Proceedings, Aachen, Germany, 445–9, 2010.

[18] Wu Y, Chen J, Ma WL, Guo L, Feng H. Virtual reality in preoperative preparation of children undergoing general anesthesia: a randomized controlled study. Anaesthesiologie. 2022;71(Supplement 2):204-11. 10.1007/s00101-022-01177-w. 35925196 10.1007/s00101-022-01177-w

[19] Deshmukh S, Murthy PS, Singh B, Contractor I. Virtual Reality as parent education tool in pre-surgical management of cleft lip and palate affected infants-A pilot study. Spec Care Dentist. 2022;42(6):606-11. 10.1111/scd.12720. 35397179 10.1111/scd.12720

[20] Park JW, Nahm FS, Kim JH, Jeon YT, Ryu JH, Han SH. The Effect of Mirroring Display of Virtual Reality Tour of the Operating Theatre on Preoperative Anxiety: A Randomized Controlled Trial. IEEE J Biomed Health Inform. 2019;23(6):2655-60. 10.1109/jbhi.2019.2892485. 30640637 10.1109/JBHI.2019.2892485

[21] Noben L, Goossens SMTA, Truijens SEM, van Berckel MMG, Perquin CW, Slooter GD, et al. A virtual reality video to improve information provision and reduce anxiety before cesarean delivery: Randomized controlled trial. JMIR Ment Heal. 2019;6(12). 10.2196/15872. 10.2196/15872 PMC6939281 31850850

[22] Page MJ, McKenzie JE, Bossuyt PM, Boutron I, Hoffmann TC, Mulrow CD, et al. The PRISMA 2020 statement: an updated guideline for reporting systematic reviews. BMJ. 2021;372:n71. 10.1136/bmj.n71. 33782057 10.1136/bmj.n71 PMC8005924

[23] Slim K, Nini E, Forestier D, Kwiatkowski F, Panis Y, Chipponi J. Methodological index for non-randomized studies (minors): development and validation of a new instrument. ANZ J Surg. 2003;73(9):712-6. 10.1046/j.1445-2197.2003.02748.x. 12956787 10.1046/j.1445-2197.2003.02748.x

[24] Aardoom JJ, Hilt AD, Woudenberg T, Chavannes NH, Atsma DE. A Preoperative Virtual Reality App for Patients Scheduled for Cardiac Catheterization: Pre-Post Questionnaire Study Examining Feasibility, Usability, and Acceptability. JMIR Cardio. 2022;6(1):e29473. 10.2196/29473. 35191839 10.2196/29473 PMC8905473

[25] Castellanos JM, Yefimov A, Dang PN. 360-Degree Virtual Reality Consultation for the Structural Heart Disease Patient. Structural Heart. 2020;4(3):230-5. 10.1080/24748706.2020.1748776.

[26] Chang SL, Kuo MJ, Lin YJ, Chen SA, Chen CT, Yang YY, et al. Virtual reality-based preprocedural education increases preparedness and satisfaction of patients about the catheter ablation of atrial fibrillation. Journal of the Chinese Medical Association : JCMA. 2021;84(7):690‐7. 10.1097/JCMA.0000000000000555. 34029219 10.1097/JCMA.0000000000000555 PMC12966114

[27] Chang SL, Kuo MJ, Lin YJ, Chen SA, Yang YY, Cheng HM, et al. Virtual reality informative aids increase residents' atrial fibrillation ablation procedures-related knowledge and patients' satisfaction. J Chin Med Assoc. 2021;84(1):25-32. 10.1097/jcma.0000000000000464. 33230060 10.1097/JCMA.0000000000000464 PMC12966046

[28] Grab M, Hundertmark F, Thierfelder N, Fairchild M, Mela P, Hagl C, et al. New perspectives in patient education for cardiac surgery using 3D-printing and virtual reality. Front Cardiovasc Med. 2023;10:1092007. 10.3389/fcvm.2023.1092007. 36937915 10.3389/fcvm.2023.1092007 PMC10020687

[29] Hermans ANL, Betz K, Verhaert DVM, den Uijl DW, Clerx K, Debie L, et al. 360degree Virtual reality to improve patient education and reduce anxiety towards atrial fibrillation ablation. Europace : European pacing, arrhythmias, and cardiac electrophysiology : journal of the working groups on cardiac pacing, arrhythmias, and cardiac cellular electrophysiology of the European Society of Cardiology. 2023. 10.1093/europace/euac246. 10.1093/europace/euac246 PMC10062331 36738261

[30] Bekelis K, Calnan D, Simmons N, Mackenzie TA, Kakoulides G. Effect of an immersive preoperative virtual reality experience on patient reported outcomes: A randomized controlled trial. Annals of Surgery. 2017;265(6):1068-73. 10.1097/SLA.0000000000002094. 27906757 10.1097/SLA.0000000000002094

[31] Perin A, Galbiati TF, Ayadi R, Gambatesa E, Orena EF, Riker NI, et al. Informed consent through 3D virtual reality: a randomized clinical trial. Acta Neurochirurgica. 2021;163(2):301-8. 10.1007/s00701-020-04303-y. 32242272 10.1007/s00701-020-04303-y

[32] Wright JM, Raghavan A, Wright CH, Shammassian B, Duan Y, Sajatovic M, et al. Back to the future: Surgical rehearsal platform technology as a means to improve surgeon-patient alliance, patient satisfaction, and resident experience. Journal of Neurosurgery. 2021;135(2):384-91. 10.3171/2020.6.JNS201865. 33096533 10.3171/2020.6.JNS201865

[33] Kapikiran G, Bulbuloglu S, Saritas S. The Effect of Video Training before Organ Transplant Surgery on Patient Satisfaction and Anxiety: head Mounted Display Effect. Clinical simulation in nursing. 2022;62:99‐106. 10.1016/j.ecns.2021.09.001.

[34] Xie L, O'Leary M, Jefferson FA, Karani R, Limfueco L, Parkhomenko E, et al. Interactive Virtual Reality Renal Models as an Educational and Preoperative Planning Tool for Laparoscopic Donor Nephrectomy. Urology. 2021;153:192-8. 10.1016/j.urology.2020.12.046. 33556447 10.1016/j.urology.2020.12.046

[35] Kwon H, Lee J, Park YS, Oh SH, Kim J. Effects of preoperative education using virtual reality on preoperative anxiety and information desire: a randomized clinical trial. J Clin Monit Comput. 2023. 10.1007/s10877-023-00988-5. 36933168 10.1007/s10877-023-00988-5

[36] Lee JJ, Klepcha M, Wong M, Dang PN, Sadrameli SS, Britz GW. The First Pilot Study of an Interactive, 360degree Augmented Reality Visualization Platform for Neurosurgical Patient Education: A Case Series. Operative neurosurgery (Hagerstown, Md). 2022;23(1):53-9. 10.1227/ons.0000000000000186. 35404334 10.1227/ons.0000000000000186

[37] Wake N, Rosenkrantz AB, Huang R, Park KU, Wysock JS, Taneja SS, et al. Patient-specific 3D printed and augmented reality kidney and prostate cancer models: impact on patient education. 3D Printing in Medicine. 2019;5(1):4. 10.1186/s41205-019-0041-3. 10.1186/s41205-019-0041-3 PMC6743040 30783869

[38] Hatzl J, Hartmann N, Bockler D, Henning D, Peters A, Meisenbacher K, et al. "Mixed Reality" in patient education prior to abdominal aortic aneurysm repair. VASA Zeitschrift fur Gefasskrankheiten. 2023. 10.1024/0301-1526/a001062. 36891667 10.1024/0301-1526/a001062

[39] House PM, Pelzl S, Furrer S, Lanz M, Simova O, Voges B, et al. Use of the mixed reality tool "VSI Patient Education" for more comprehensible and imaginable patient educations before epilepsy surgery and stereotactic implantation of DBS or stereo-EEG electrodes. Epilepsy research. 2019;159:106247. 10.1016/j.eplepsyres.2019.106247. 31794952 10.1016/j.eplepsyres.2019.106247

[40] Williams MA, McVeigh J, Handa AI, Lee R. Augmented reality in surgical training: a systematic review. Postgrad Med J. 2020;96(1139):537-42. 10.1136/postgradmedj-2020-137600. 32229513 10.1136/postgradmedj-2020-137600

[41] Urlings J, Sezer S, Ter Laan M, Bartels R, Maal T, Boogaarts J, et al. The role and effectiveness of augmented reality in patient education: A systematic review of the literature. Patient Educ Couns. 2022;105(7):1917-27. 10.1016/j.pec.2022.03.005. 35341611 10.1016/j.pec.2022.03.005

[42] Lan L, Mao RQ, Qiu RY, Kay J, de Sa D. Immersive Virtual Reality for Patient-Specific Preoperative Planning: A Systematic Review. Surg Innov. 2023;30(1):109-22. 10.1177/15533506221143235. 36448920 10.1177/15533506221143235 PMC9925905

[43] Dicpinigaitis AJ, Li B, Ogulnick J, McIntyre MK, Bowers C. Evaluating the Impact of Neurosurgical Educational Interventions on Patient Knowledge and Satisfaction: A Systematic Review of the Literature. World Neurosurg. 2021;147:70-8. 10.1016/j.wneu.2020.11.144. 33276172 10.1016/j.wneu.2020.11.144

[44] Bollen E, Awad L, Langridge B, Butler PEM. The intraoperative use of augmented and mixed reality technology to improve surgical outcomes: A systematic review. Int J Med Robot. 2022;18(6):e2450. 10.1002/rcs.2450. 35971649 10.1002/rcs.2450

[45] Wu X, Liu R, Yu J, Xu S, Yang C, Yang S, et al. Mixed Reality Technology Launches in Orthopedic Surgery for Comprehensive Preoperative Management of Complicated Cervical Fractures. Surg Innov. 2018;25(4):421-2. 10.1177/1553350618761758. 30012077 10.1177/1553350618761758

[46] Yoshida S, Taniguchi N, Moriyama S, Matsuoka Y, Saito K, Fujii Y. Application of virtual reality in patient explanation of magnetic resonance imaging-ultrasound fusion prostate biopsy. Int J Urol. 2020;27(5):471-2. 10.1111/iju.14204. 32115774 10.1111/iju.14204

[47] Shepherd T, Trinder M, Theophilus M. Does virtual reality in the preoperative setting for colorectal cancer surgery improve patient understanding? A randomized pilot study. ANZ J Surg. 2024;94(3):391-6. 10.1111/ans.18787. 37994285 10.1111/ans.18787

